# Treatment of Malarial Coma

**Published:** 1882-05-20

**Authors:** Arch. Dixon

**Affiliations:** Henderson, Kentucky


### TREATMENT OF MALARIAL COMA.

BY ARCH. DIXON, M. D., OF KENTUCKY.

In a recent number of the Virginia Medical Monthly, there appeared an article of great interest and practical value to those who live in malarial regions, on "hypodermic use of quinia in certain fevers," by Dr. W. T. Sawyer, of Alabama. I wish to add my testimony to the efficacy of the hypodermic use of quinia in certain cases which every physician who practices in the South and West will occasionally, and during some seasons, often meet with. The year 1878 was typical in this respect, the ordinary malarial fevers of our climate assuming a pernicious character, and we had many cases which were appropriately called by the late Dr. John L. Cook, of this place, "*Malarial Coma.*" The history of these cases was generally that of an ordinary intermittent, which had failed to respond to the usual anti-periodic treatment given by the family, the paroxysms presenting nothing denoting gravity, until after one or two chills, when the case assumed a more serious character. There is frequent nausea and an inclination to drowsiness, gradually increasing until there is complete coma; the face is suffused; the pupils contracted and irresponsive to light; the pulse is small and rapid; in some cases slow and full; breathing hurried and shallow, occasionally stertorous and not infrequently of the "Cheyne-Stokes" character. Temperature runs up to 165-7.

There is loss of voluntary motion, with complete banishment of consciousness. In short, there is a condition of general congestion, more especially of the brain and lungs. The situation is alarming, and the doctor is called in haste. The gravity of the case is taken in at a glance, and the conclusion arrived at, that something must be done, and be done quickly.

The following typical case, selected from a number of others, similarly treated, will illustrate what was done by the writer, and with such success as to convince him that the treatment was good.

On Sunday, Sept. 15th, 1878, was called in haste to see Wm. McC., and was informed by the messenger, his father, that his son had brain fever; that he had had a spasm, and was now out of his head. I found the patient, a stout young man, aged 19 years, in a deep coma, pupils markedly contracted and irresponsive to light; pulse 130, small and weak; breathing stertorous; temperature 106. I gathered from his mother, that he had been taken with a chill about 11 o'clock on Friday morning; his fever had not been higher than that which ordinarily follows a chill, but that he had complained much of headache. On Saturday he felt almost as well as usual, and expressed a desire to go to work, but had been dissuaded by her. Friday night a cathartic dose of calomel and soda was taken, which acted well, and had been followed on Saturday morning by twenty-one grains of cinchonidia, given in seven grain doses, at intervals of three hours. At 5 a. m., Sunday, took seven grains of cinchonidia, but expressed himself as feeling too badly to get up. He complained of headache and nausea. At 8 o'clock he was to take another powder, and she found him with fever and so drowsy that it was difficult to arouse him; he, however, took the medicine and almost immediately went off to sleep again. At 10 o'clock he had a slight convulsion, which had passed off after he vomited some blood. At 11 o'clock I found him in the condition described. To give anything by the mouth was out of the question, as the patient could neither swallow, nor could I afford to await the slow process of absorption by the stomach, which in all probability might not take place at all. There was total loss of all voluntary motion, and there was a cataleptoid condition present, the limbs remaining in whatever position they were placed.

A subcutaneous injection of morphia 1-8 gr., and atropia 1-120 gr., was made at once, and as soon as possible, a large sheet immersed in cold water was spread over a blanket stretched upon the floor, on this my patient was placed after having been stripped of all his clothing. By this time the hypodermic injection of morphia and atropia had stimulated both respiration and circulation, the pulse was stronger and fuller, and the breathing easier. He was now wrapped in the sheet and cold water poured upon him for ten minutes; cold applications were first made to the head, by means of towels wrung out in cold water; meanwhile, I had hastily dissolved, gr. xx. of quinia bi-sulph. in ℥ ij. of boiling water; one-third of this was administered *hypodermically* before removing the patient from the wet pack. He was now taken up and placed between blankets on the bed, without wiping off his body. The thermometer showed a reduction of temperature from 106 to 102, and there were signs of returning consciousness. The remaining two-thirds of quinia solution were given at intervals of fifteen minutes, the patient being rational before the last injection was made. Ten grains of quinia were ordered to be given at intervals of two hours, that evening, till thirty grains were taken; the same to be repeated the following morning. My patient had no further trouble, and soon grew strong upon a tonic pill of iron, quinia and arsenic.

There can be no doubt that quinia is the one thing needful in these cases, and used hypodermically, it is simply invaluable. My attention was called to this method of using it by an article in the London Lancet, in 1876, by J. B. Scriven, Civil Surgeon at Lahore. The solution was made with tartaric acid, one in three. Mr. Scriven had used it in a great many cases, and advised the introduction of the nozzle of the syringe so that the apparatus, by which the quinia escapes into the cellular tissue, shall be turned *away* from the skin, in order to avoid abscess, ulceration or sloughing. The injection should be thrown slowly *into the cellular tissue*, and not immediately beneath the skin. This method has been highly satisfactory in my hands, and in no instanee has any bad effect followed its use. In many cases the wet pack is an admirable adjunct, and in no way can temperature be reduced so rapidly save by the cold bath. It is hardly necessary to say that caution should be exercised, both in the use of the bath and pack, for you may pass a point beyond which reaction fails to take place. In cases of this character, pulmonary, hepatic and gastric complications, you cannot afford to waste valuable remedies directed against them, and the physician who awaits the action of remedies given by the mouth or rectum, will too often find the life of his patient slipping through his hands; for there is present in almost every case, a condition of irritability, both gastric and intestinal, which precludes the probability of the medicine being retained, and if retained, the intense congestion prevents absorption, and notwithstanding large doses of quinia have been taken, you watch anxiously and in vain for its characteristic action, your patient growing hourly worse, until he "crosses over the river," to join the innumerable throng of those who have gone before. The indications in such cases are to reduce temperature as rapidly as possible, using the wet pack boldly, yet cautiously; *give quinia hypodermically*, repeating at short intervals until its physiological action is manifest, stimulate your patient and you will have the satistion of seeing your efforts crowned with success.

*Henderson, Ky., April 35, 1882.*

